# Environmental Tobacco Smoke Exposure during Intrauterine Period, Promotes Caspase Dependent and Independent DNA Fragmentation in Sertoli-Germ Cells

**DOI:** 10.1155/2014/170124

**Published:** 2014-03-12

**Authors:** Beril Yüksel, Sevtap Kilic, Nese Lortlar, Nicel Tasdemir, Semra Sertyel, Yesim Bardakci, Tarik Aksu, Sertaç Batioglu

**Affiliations:** ^1^Dumlupinar University, School of Medicine, Department of Obstetrics and Gynecology, 43000 Kutahya, Turkey; ^2^Dr. Zekai Tahir BURAK Women Health, Education and Research Hospital, Department of Reproductive Endocrinology and IVF, Cebeci, 06230 Ankara, Turkey; ^3^Gazi University, Faculty of Medicine, NanoMedicine Research Laboratory, Golbasi, 06810 Ankara, Turkey; ^4^Namik Kemal University, Faculty of Medicine, 59000 Tekirdag, Turkey; ^5^HRS Women Health and IVF Center, Cankaya, 06700 Ankara, Turkey; ^6^Samsun 19 Mayıs University, Faculty of Medicine, 55220 Samsun, Turkey

## Abstract

*Objectives*. To investigate the effect of cigarette smoke exposure during intrauterine period on neonatal rat testis. *Methods*. Twenty-five rats were randomized to be exposed to cigarette smoke with the Walton Smoking Machine or to room air during their pregnancies. The newborn male rats (*n* = 21) were grouped as group 1 (*n* = 15) which were exposed to cigarette smoke during intrauterine life and group 2 (*n* = 6) which were exposed to room air during intrauterine life. The orchiectomy materials were analyzed with TUNEL immunofluorescent staining for detection of DNA damage. To detect apoptosis, immunohistochemical analyses with caspase-3 were performed. Primary outcomes were apoptotic index and immunohistochemical scores (HSCORES); secondary outcomes were Sertoli-cell count and birth-weight of rats. *Results*. Sertoli cell apoptosis was increased in group 1 (HSCORE = 210.6 ± 41.9) when compared to group 2 (HSCORE = 100.0 ± 17.8) (*P* = 0.001). Sertoli cell count was decreased in group 1 (*P* = 0.043). The HSCORE for the germ cells was calculated as 214.0 ± 46.2 in group 1 and 93.3 ± 10.3 in group 2 (*P* = 0.001) referring to an increased germ cell apoptosis in group 1. The apoptotic indexes for group 1 were 49.6 ± 9.57 and 29.98 ± 2.34 for group 2 (*P* = 0.001). The immunofluorescent technique demonstrated increased DNA damage in seminiferous epithelium in group 1. *Conclusions*. Intrauterine exposure to cigarette smoke adversely affects neonatal testicular structuring and diminishes testicular reserve.

## 1. Introduction

Apoptosis or programmed cell death plays an important role in the homeostasis and normal function of all tissues. It is morphologically characterized by disruption of the cell skeleton, cell shrinkage, membrane blebbing, nuclear condensation, cell disruption into small membrane-enclosed fragments, and phagocytosis by neighbouring cells [1].

DNA fragmentation is a key feature of programmed cell death. DNA cleavage during apoptosis occurs at sites between nucleosomes. It is characterized by the activation of endogenous endonucleases with subsequent cleavage of chromatin DNA into internucleosomal fragments [2].

The induction and execution of apoptosis require the cooperation of a series of molecules. Among them, the caspase-cascade signaling system is vital in the process of apoptosis [3]. Generally, the caspase family proteases can be activated through three pathways, mediated by either mitochondrion/cytochrome-C, cell surface receptors, or endoplasmic reticulum [4–6]. Caspase-3, the major effector caspase, is synthesized as an inactive proenzyme and processed during apoptosis into its active form. In this study, caspase-3 was selected because it represents the point of intersection of extrinsic, intrinsic, and mitochondrial pathways [7].

Smoking is one of the most important health issues and the most wide-spread habituation worldwide. It is well established that smoking is associated with a significant reduction in sperm concentration and quality [8]. Recent studies have also demonstrated that the apoptotic processes may participate in abnormal spermatogenesis in nonobstructive azoospermia, such as maturation arrest and Sertoli cell-only syndrome [9, 10].

The rise of tobacco use among pregnant women has promoted concern over the potential adverse effects on their offspring. Maternal smoking during pregnancy is well known to be associated with a variety of adverse neonatal outcomes. But the effects of intrauterine exposure to cigarette smoke on newborns' gonads are also intriguing. Unfortunately there are only a few studies related to this subject in the literature. Therefore, in this study, we aimed to investigate the effect of cigarette smoke exposure during intrauterine period on neonatal rat testis.

## 2. Material and Method

Before starting the study, an approval from local educational research committee was taken. The research was consistent with* World Medical Association Declaration of Helsinki* and the* Guide for the Care and Use of Laboratory Animals* of the Institute for Laboratory Animal Research of the National Research Council.

**Figure 1:**
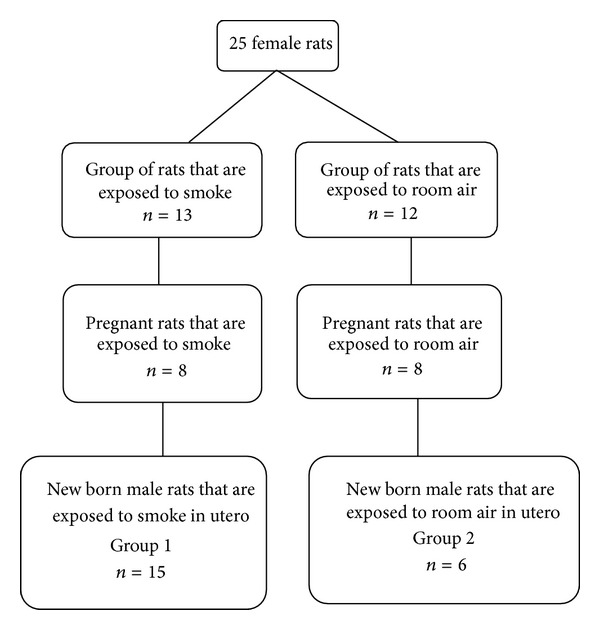
Consort flow diagram.

A prospective, randomized and experimental study was conducted with 25 female Wistar albino rats. Consort guidelines were followed through the study (Figure 1).

The rats were randomized to two groups (*n* = 13, *n* = 12), either to be exposed to room air or cigarette smoke with the Walton Smoking Machine (Process and Instruments Corp., Brooklyn, NY). The rats were exposed to 2R4F Kentucky Tobacco Research and Development Center reference cigarettes (9.7 mg of tar, 0.85 mg of nicotine, and 11.7 mg of total particulate matter) one hour twice a day for a total of 10 cigarettes per day, initiating from proestrous period and during their pregnancies. Each cycle on the machine included one puff of cigarette smoke of 2-second duration, followed by a 28-second hold period, for a smoke exposure time of 30 seconds per cycle. This was followed by a 30-second purge of fresh air before the second puff is taken to repeat the cycle. The remaining rats were exposed to room air with the same machine and served as controls. The rats were mated with male rats. Next morning, the female rats that had spermatozoa on their vaginal smears were accepted pregnant (*n* = 16). The remaining rats were excluded from the study.

The newborn male rats (*n* = 21) were categorized as group 1 and group 2. Group 1 included 15 newborn male rats that were exposed to cigarette smoke during their intrauterine life. Group 2 included 6 newborn male rats that were exposed to room air during their intrauterine life. The birth weights of all rats were recorded.

The rats were sacrificed at the end of their first week of life. Bilateral orchiectomy materials were fixed in Bouin solution.

### 2.1. Light Microscopy

After fixation with 10% formalin, the tissues were washed under running tap water for 24 hours and dehydrated with 50, 60, 70, 80, 90, 96, and 100% concentrated ethanol. The specimens were then laid in a 1 : 1 ratio of immersion oil and absolute alcohol for 1 hour, followed by immersion oil overnight for transparency. After the application of xylol, the specimens were made into paraffin blocks using a 1 : 1 xylol and paraffin mixture for 1 hour and paraffin for 6 hours in an incubator. 4 *μ*m thick sections were dehydrated and dyed with Hematoxylin Harris (Biostain Code: RR SP67-D/Biostain Ready Reagents Ltd., Unit 2A, Arrow Trading Estate, Manchester).

### 2.2. Immunohistochemistry

To detect apoptosis, immunohistochemical analyses with anti-caspase-3 primary antibodies were performed. After embedding in paraffin, 4 *μ*m thick sections were taken onto poly-l-lysine coated slides. After incubating at 37°C overnight and 60°C for an hour, the sections were dewaxed in xylene for 15 minutes twice and rehydrated with descendent ethanol gradient for 10 minutes and distilled water for 5 minutes. After dewaxing and rehydration with xylene, alcohol, and distilled water, respectively, the slides were taken into microwave irradiation containing 0.1 M citrate buffer (catalog number AP-9003-500, Lot CT14070, LabVision Corporation, Fremont, CA, USA) for 5 minutes at 650 W and for 3 × 5 minutes at 550 W.

After 20 minutes at room temperature, the sections were encircled with a PAP Pen (Super PAP Pen, PN IM3580, Beckman Coulter Company, France). By a new washing with distilled water and then with phosphate-buffered saline (PBS, Lot 0446B002, LabVision Corporation, Fremont, CA, USA), endogenous peroxidase activity was blocked by using 3% hydrogen peroxide (catalog number TA-125-HP, Thermo Scientific, Fremont, CA, USA) for 20 minutes. After washing with PBS, ultra V block (catalog number TA-125-UB, Thermo Scientific, Fremont, CA, USA) was applied for 5 minutes.

After a 1 hour application of primary antibodies (caspase-3; catalog number RB-1197, Thermo Scientific, Fremont, CA, USA) diluted (1 : 100 concentration) with antibody diluents (catalog number TA-125-UD Thermo Scientific, Fremont, CA, USA), the samples were washed with PBS again. Then secondary antibody, biotinylated goat antipolyvalent (catalog number TP-125-BN, Thermo Scientific, Fremont, CA, USA) was applied for 20 minutes. The sections were washed with PBS and treated with streptavidin peroxidase (TP-125-HR, Thermo Scientific, Fremont, CA, USA) for 20 minutes. By a new washing with PBS, finally, the specimens were placed for 10 minutes in DAB (3,3′ diaminobenzidine) (Thermo Scientific, catalog number TA-125-HD, Fremont, CA, USA) and stained with Hematoxylin Harris (Biostain Code: RR SP67-D/Biostain Ready Reagents Ltd., Unit 2A, Arrow Trading Estate, Manchester).

The slides were covered with xylol-based mounting, evaluated under Leica DM 4000 B light microscope (Wetzlar, Germany), and photographed with Leica QWin ProV 3.4.0 (Calidris and SoftHard Technology Ltd., Switzerland).

### 2.3. Immunofluorescence

In situ terminal deoxynucleotidyl transferase-mediated dUTP nick end-labeling (TUNEL) immunofluorescent staining was used for detection of DNA damage. The formalin fixed, paraffin-embedded tissues were dewaxed in xylene and rehydrated through a graded series of ethanol and double-distilled water. Then they were placed in a plastic jar and incubated for 5 minutes in 350 W microwave irradiation containing 200 mL 0.1 M citrate buffer, pH 6.0. After rinsing the slides twice with PBS, 50 *μ*L TUNEL reaction mixture (catalog number 11 684 795 910, Roche Diagnostics, Roche Diagnostics Gmbh, Roche Applied Science, Mannheim, Germany) per sample was added. Then the samples were incubated for 60 minutes at 37°C in a humidified atmosphere in the dark. Finally, the slides were rinsed three times with PBS and analyzed under a fluorescence microscope with a wavelength in the range of 450–500 nm (e.g., 488 nm) and detection in the range of 515–565 nm (green).

### 2.4. Apoptotic Index

At least 500 cells were counted on each slide. The TUNEL-positive cells which also showed apoptotic nuclear morphology were defined as apoptotic and the percentage of these cells to total cell number was recorded as apoptotic index [11].

### 2.5. Immunohistochemical Scoring

An immunohistochemical score (HSCORE) was calculated as the sum of the percentages of positively stained epithelial cells multiplied by the weighted intensity of staining: HSCORE = ∑*Pi*(*I* + 1), where “*I*” represents staining intensity (0 = no expression, 1 = mild, 2 = moderate, and 3 = intense) and “*Pi*” is the percentage of stained cells for each intensity [12].

### 2.6. Sertoli Cell Count

The numbers of Sertoli cells were quantified in cross sections of seminiferous tubules stained by H and E. The number of Sertoli cells per tubule was computed in 30 tubules per animal using a light binocular microscope at ×400 [13]. Only Sertoli cells exhibiting typical morphological nuclear features and evident nucleolus were quantified.

### 2.7. Statistical Analysis

SPSS 15.0 for windows was used for the statistical analysis. Whether the distributions of variables were normal or not was determined by means of the Kolmogorov Smirnov test. Student's *t*-test was used for the comparison of normal distributed variables. A *P* value of less than 0.05 was considered statistically significant.

## 3. Results

For the comparison of the groups, the primary outcomes were defined as apoptotic index and HSCORES and the secondary outcomes were defined as Sertoli cell count and birth weights of newborn male rats.

**Figure 2:**
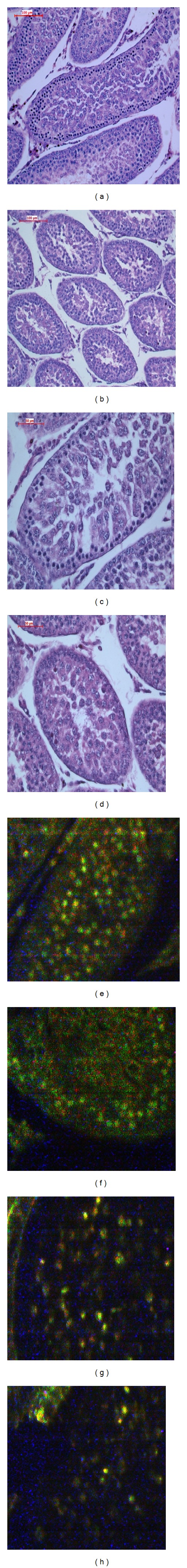
(a–d) The light microscopic view of the tissues. The cells showing pyknotic nuclei with nuclear condensation and condensed eosinophilic cytoplasm were scored as apoptotic cells. (a–c) The light microscopic view group 1. (b–d) The light microscopic view group 2. (e–h) TUNEL immunofluorescent staining for the detection of DNA damage. (e-f) Testicular tissues of group 1. (g-h) Testicular tissues of group 2.

When Hematoxylin-Eosin stain preparation was compared, the cells showing apoptotic morphology with pyknotic nuclei, nuclear condensation, and condensed eosinophilic cytoplasm were more remarkable in group 1 (Figure 2). The immunofluorescent technique also demonstrated increased DNA damage in seminiferous epithelium in group 1 (Figure 2).

For the quantitative analysis, the apoptotic indexes were compared between groups. It was 49.61 ± 9.57 for intrauterine smoke exposure group (group 1, *n* = 15) and it was 29.98 ± 2.34 for intrauterine room air exposure group (group 2, *n* = 6). The difference was statistically significant (*P* < 0.001).

**Figure 3:**
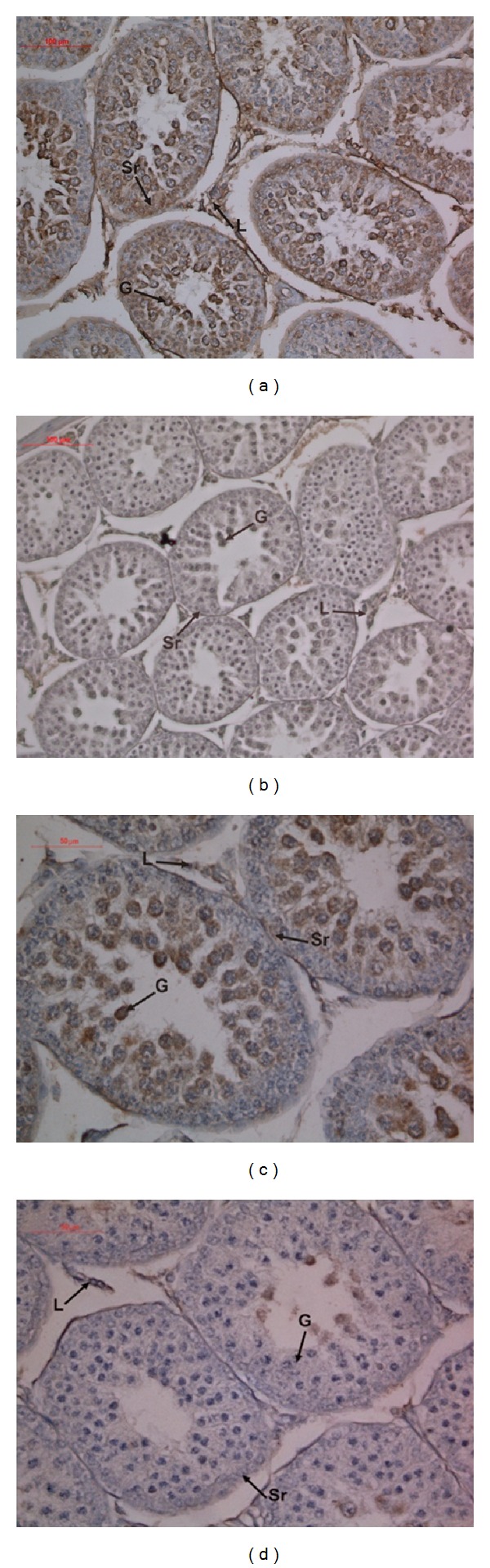
Immunohistochemical analyses with caspase-3 for the detection of apoptosis. (a–c) Testicular tissues of group 1. (b–d) Testicular tissues of group 2.

When the slides were evaluated immunohistochemically for the detection of caspase-3 activity, cytoplasmic and nuclear caspase-3 immunostaining was detected both in the germ and Sertoli cells in group 1 (Figure 3).

For the quantitative analysis, HSCORES were calculated. There was a significant increase in the incidence of Sertoli cell apoptosis, detected by caspase-3 immunostaining, in group 1 (HSCORE = 210.6 ± 41.9) when compared to group 2 (HSCORE = 100.0 ± 17.8) (*P* = 0.001). There was also a significant increase in the incidence of germ cell apoptosis in group 1 (HSCORE value for group 1 is 214.0 ± 46.26 and HSCORE value for group 2 is 93.33 ± 10.32, *P* < 0.001).

When the Sertoli cell counts were determined, the median value for group 1 was 9.0 (6–11) and 11.5 (10–13) for group 2. The difference was statistically significant (*P* = 0.043).

When the birth weights of newborn male rats were analyzed, the mean birth weight of group 1 was significantly lower than group 2 (3.08 ± 0.26 versus 4.47 ± 0.40, *P* < 0.001).

## 4. Discussion

Spermatogenesis is a dynamic process of cell differentiation characterized by mitotic and meiotic divisions, which transform the stem spermatogonia into final mature spermatozoa. Throughout this process of development and differentiation, a considerable number of germ cells die due to apoptosis to control the overproduction of male gametes [14].

It is well known that the paracrine signaling between Sertoli cells regulates the process of germ cell death. In the presence of exogenous stimulants, such as cadmium, nicotine, lead, and benzopiren that adversely affect testicular function, nonphysiologic apoptosis and DNA fractures can occur which leads to impaired spermatogenesis and infertility.

In a study by Lin et al. in 1997 increased apoptosis was detected in testis biopsy specimens of azoospermic and severe oligozoospermic patients and this finding was suggested as one of the causes of male infertility [15]. In the following years numerous investigations were composed for the effects of cigarette smoking on impaired spermatogenesis and increased apoptosis has been found to occur during maturation arrest, hypospermatogenesis, and Sertoli cell only syndrome [9, 16, 17].

Despite the well-known effects of cigarette smoke on the reproductive performance of the individuals, we have a limited data for its effects on the newborns gonads. Epidemiological studies have demonstrated that sperm production and testicular volumes are dose dependently reduced in the adult sons of mothers who smoked during pregnancy [18]. Other studies showed that sons exposed to maternal smoking in uteri have a reduction in sperm concentration of between 20 and 48% in comparison to unexposed ones [19, 20].

In our study we determined an increased apoptosis and DNA damage, in the gonads of the newborns of the study group. Abnormality of the oxidant-antioxidant balance may be one of the mechanisms leading to testicular tissue damage following chronic inhalation of cigarette smoke [21]. Cigarette smoke contains agents acting on the aryl hydrocarbon receptor (AHR). It is suggested that activation of AHR by environmental toxicants and AHR-induced apoptotic pathways may be another mechanism [22]. Activation of coagulation in the intervillous space in addition to vasoconstrictor effects of carbon monoxide and nicotine could reduce the oxygen transport to the fetus which can also cause an increase in apoptosis [23].

It is well known that tobacco use is associated with fetal growth restriction and low birth weight. In our study we detected lower birth weights in the in uteri smoke exposed group which concurs well with the literature. In a study by Wang et al. smoking during pregnancy is shown to result in an average of 377 grams of decrease in birth weight [24]. In another study when the placentas of smoker mothers were analyzed, premature aging and degenerative changes were detected and it was suggested to be the cause of low birth weight newborns [25].

Despite remarkable advances in the treatment of severe male factor infertility, significant numbers of men cannot be effectively treated because of deficiencies in our understanding of testicular failure pathophysiology. Therefore, the mechanisms responsible for abnormal spermatogenesis should be clarified in order to develop and improve treatment options.

Numerous investigations were composed for the effects of cigarette smoking on impaired spermatogenesis. But apart from those studies, this study evaluated the effect of exposure to cigarette smoke in the intrauterine period which, to our knowledge, was not undertaken before.

## 5. Conclusion

Chronic cigarette smoke is shown to induce apoptosis in the rat testis [26]. And in our previous report, we showed that tobacco smoke exposure during intrauterine period promotes granulosa cell apoptosis in newborn rat ovaries [27]. Here in this study, using immunohistochemical and immunofluorescent method, we demonstrated significantly increased apoptosis and DNA damage in the newborn male gonads of intrauterine cigarette smoke exposed group and we hope that these data will be an inspiration for the future studies.
